# *Nigella damascena* L. Essential Oil—A Valuable Source of β-Elemene for Antimicrobial Testing

**DOI:** 10.3390/molecules23020256

**Published:** 2018-01-28

**Authors:** Elwira Sieniawska, Rafal Sawicki, Joanna Golus, Marta Swatko-Ossor, Grazyna Ginalska, Krystyna Skalicka-Wozniak

**Affiliations:** 1 Department of Pharmacognosy with Medicinal Plant Unit, Medical University of Lublin, Chodzki 1 Street, 20-093 Lublin, Poland; kskalicka@pharmacognosy.org; 2 Department of Biochemistry and Biotechnology, Medical University of Lublin, Chodzki 1 Street, 20-093 Lublin, Poland; joanna.golus@umlub.pl (J.G.); martaswatkoossor@umlub.pl (M.S.-O.); grazynaginalska@umlub.pl (G.G.)

**Keywords:** GC-MS, sesquiterpenoids, ranunculaceae, essential oil, mycobacteria, tuberculosis, countercurrent separation, MIC

## Abstract

The most commonly used plant source of β-elemene is *Curcuma wenyujin* Y. H. Chen & C. Ling (syn. of *Curcuma aromatic* Salisb.) with its content in supercritical CO_2_ extract up to 27.83%. However, the other rich source of this compound is *Nigella damascena* L. essential oil, in which β-elemene accounts for 47%. In this work, the effective protocol for preparative isolation of β-elemene from a new source—*N. damascena* essential oil—using high performance counter-current chromatography HPCCC was elaborated. Furthermore, since sesquiterpens are known as potent antimicrobials, the need for finding new agents designed to combat multi-drug resistant strains was addressed and the purified target compound and the essential oil were tested for its activity against a panel of Gram-positive and Gram-negative bacteria, fungi, and mycobacterial strains. The application of the mixture of petroleum ether, acetonitrile, and acetone in the ratio 2:1.5:0.5 (*v/v*) in the reversed phase mode yielded β-elemene with high purity in 70 min. The results obtained for antimicrobial assay clearly indicated that *N. damascena* essential oil and isolated β-elemene exert action against *Mycobacterium tuberculosis* strain H37Ra.

## 1. Introduction

β-Elemene, a natural sesquiterpene and its isomers were isolated for the first time in 1994 from dry curcuma rhizome, and until now rhizome of curcuma (species of *C. Wenyujin* Y. H. Chen & C. Ling, *C. phaeocaulis* Valeton, and *C. kwangsiensis* S. G. Lee & C. F. Liang, Zingiberaceae) is the only plant source that has been used in elemene processing industry [1]. The described content of β-elemene in extract of rhizome curcuma processed during industrial supercritical CO_2_ extraction is up to 27.83%; however, there are several plant species more abundant in β-elemene or its isomers in their essential oils (*Nigella damascena* L., *Magnolia figo* (Lour.) DC, *Alisma plantago-aquatica subsp. orientale* (Sam.) Sam. and *Solidago decurrens* Lour). Among them, *N. damascena* essential oil is the most promising source in which β-elemene can reach up to 73% [1].

Techniques previously used for β-elemene isolation include classical column chromatography: silica gel column chromatography [2], silver ion coordination chromatography [3,4,5], or HP20 macroporous resin chromatography [5] with further purification by means of preparative high performance liquid chromatography [5]. Also, preparative gas chromatography with dimethylpolysiloxane or enantioselective cyclodextrin columns were applied [6]. Modern techniques based on the principle of liquid-liquid extraction (in which no losses of sample occurs), like high-performance centrifugal partition chromatography (HPCPC) [7] or high-speed counter-current chromatography (HSCCC) [8], were used to obtain β-elemene from plant material/essential oil as well. In our study the other modern technique based on the principle of liquid-liquid extraction, high performance counter-current chromatography (HPCCC), was applied, because it enables the achievement of a higher *g* level, and thus higher efficiency, compared to high-speed counter-current chromatography. Furthermore, the rapid purification can be obtained due to providing good retention of a high amount of the stationary phase and possibility to use higher flow rate of the mobile phase [9,10]. To our knowledge, this technique was not previously used for β-elemene isolation [10,11]; however, there are few papers describing successful purification of terpenoids from essential oils with application of HPCCC [12,13,14].

There is little information regarding antimicrobial action of β-elemene. What is more, the increasing incidence of multi drug resistance of microorganisms to different antibiotics and chemotherapeutics is a strong reason for searching for new active agents [15]. Sesquiterpenes isolated from essential oils are among compounds with promising antimicrobial activity [16,17]. Also, β-elemene is natural sesquiterpene; therefore, it is worth to check the antimicrobial activity of this compound against different pathogens including mycobacteria.

Hence, in this work, we aimed to elaborate the effective protocol for preparative isolation of β-elemene from a new plant source—*N. damascena* essential oil—using HPCCC technique, and to check its antimicrobial action and compare it with activity of crude essential oil against panel of Gram-positive, Gram-negative bacteria, fungi, and mycobacterial strains.

## 2. Results

### 2.1. Chemical Composition of N. damascena Essential Oil

**Figure 1:**
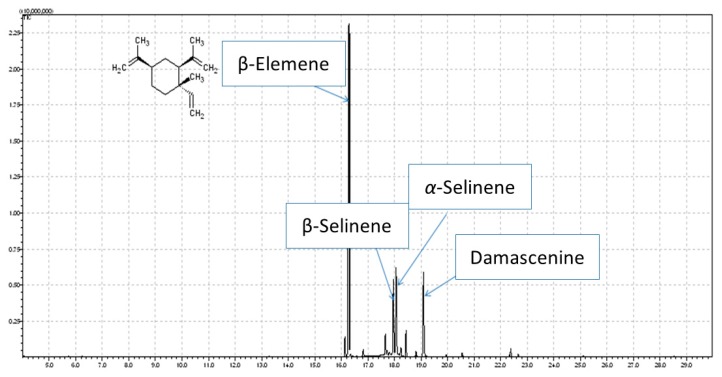
GC-MS chromatogram of *Nigella damascena* essential oil and chemical structure of β-elemene.

GC-MS analysis was used to confirm a proper selection of source material for β-elemene isolation. *N. damascena* essential oil (EO) obtained via hydrodistillation constituted 0.436% of dry plant material. 29 compounds representing over 99% of the total EO were identified (Table 1), with β-elemene being the main ingredient and accounting for 47.37% in the EO (Figure 1). The other compounds present in the amount above 10% and listed according to the elution time were β-selinene, *α*-selinene, and damascenine. Based on these data, we subjected *N. damascena* EO to HPCCC separation as optimal source for β-elemene isolation.

### 2.2. Isolation of β-Elemene

The selection of solvent systems used for determination of K*_β_*_-elemene_ was based on the previous work of Dang et al., who separated β-elemene along with five other volatiles from *C. wenyujin* essential oil [7]. The K*_β_*_-elemene_ values obtained in our study (Table 2) differed from this reported previously (systems 1 and 2); what is more, heptane/acetonitrile/ethyl acetate in the ratio 2:1:1 became a single phase above 23 °C. The additional variants of mixture of petroleum ether/acetonitrile/acetone (systems 3 and 4) gave satisfactory K*_β_*_-elemene_ values; however, the problem with system stability over 23 °C appeared again in case of system 3. The final separation was carried out in a mixture of petroleum ether/acetonitrile/acetone in the ratio 2:1.5:0.5, with a good retention of stationary phase (78%). β-elemene was eluted between 55–70 min (Supplementary Materials: Figure S1), and its purity in the fractions ranged from 87 to 96%. The optimization of the sample mass revealed that the best purity (96%) and yield (80%) was obtained when 100 mg of essential oil was used (Table 3). Compounds other than β-elemene and damascenine (eluted in first ten minutes of the run) were retained in the column as having higher K values (data not shown).

### 2.3. Identification of Isolated β-Elemene

Spectroscopic data: MS (EI, 70 eV) *m/z* (%): 81 (100); 93 (95); 107 (70); 67 (60); 121 (45); 147 (45); 56 (35); 121 (35); ^1^H NMR (300 MHz, CDCl_3_), δ: 5.86–5.80 (1H, dd, *J* = 11.0, 17.4), 4.93–4.89 (2H, m), 4.83–4.82 (1H, t, *J* = 1.6), 4.73–4.71 (2H, m), 4.60 (1H, bs), 2.04–2.00 (1H, m), 1.96–1.92 (1H, m), 1.75 (3H, s), 1.72 (3H, s), 1.62–1.43 (6H, m), 1.01 (3H, s). ^13^C NMR (9300 MHz, CDCl_3_), δ: 150.4, 150.3, 147.7, 112.1, 109.8, 108.2, 52.7, 45.7, 39.9, 39.8, 32.9, 26.8, 24.8, 21.1, 16.6

Chromatographic profiles (Supplementary Materials: Figure S2A,B) and spectroscopic data (Supplementary Materials: Figure S2C; Figures S3 and S4) matched the molecular structure of β-elemene, which was also in accordance with previous reports [5].

### 2.4. Antimicrobial Testing

The antimicrobial activity of isolated β-elemene, as well as *N. damascena* essential oil, was checked against standard panel of microorganisms including Gram-positive, Gram-negative bacteria, fungi, and mycobacterial strains. The MIC reading was normalized against controls with 1% DMSO (with bacteria/yeasts) and blanks (without bacteria/yeasts), and compared with the positive control drugs. Normalization means that antibiotics with MIC values known for the tested set of strains were used. The obtained control values were within the reference ranges, which proves that the MIC test was performed correctly.

The control with DMSO solvent confirmed that there is no influence of the solvent itself on the growth of tested organisms; hence, the obtained results are not false positive. Our findings regarding the use of DMSO solvent are supported by earlier study performed by O’Neill et al., who studied the minimal inhibitory effect of this solvent against *M. tuberculosis* in microplate resazurin assay [18] and by Wadhwani et al. who evaluated the influence of different solvents, including DMSO, on a growth of several bacteria species [19]. Both research teams found the 2% DMSO concentration is maximal acceptable with regard to the compromise between solubility of tested compounds and providing sufficient bacteria growth. In our study, 1% DMSO was used, because the solubility of studied samples was not problematic in this concentration.

No activity of β-elemene, as well as *N. damascena* essential oil, was observed against *Staphylococcus aureus*, *Staphylococcus epidermidis*, *Escherichia coli*, *Pseudomonas aeruginosa*, *Candida albicans*, *Candida parapsilosis,* and *Mycobacterium smegmatis* (MIC > 1000 μg/mL); however, the medium activity was noticed for both samples against *M. tuberculosis* (MIC value of 128 μg/mL and 256 μg/mL for β-elemene and EO, respectively). The obtained results clearly indicate that *N. damascena* EO and β-elemene itself exert moderate action against *M. tuberculosis* H37Ra strain (Table 4).

## 3. Discussion

### 3.1. N. damascena Essential Oil Is a New, Rich Source for β-Elemene Isolation

EO used in this study was obtained from seeds of *N. damascena* cultivated in Poland, and it contained 47% of β-elemene. Higher content of β-elemene, reaching up to 73%, was reported previously by D’Antuono et al. [20] and by Moretti et al. [21]. However, results similar to ours were obtained by Fico et al. [22] and by Wajs et al. [23], who also found β-elemene content in the essential oil obtained from *N. damascena* seeds equal to 50% and 59%, respectively. The chemical composition of essential oils and plant extracts is influenced by different factors, among which climate, growing conditions, or harvest time are the most studied [24]. The essential oil used in our study was hydrodistilled form seeds obtained from market in Poland. Wajs et al. also studied polish seeds purchased from the market [23]. However, Fico, who found similar content of β-elemene (49%), studied seeds from plants grown in Italy. Interestingly, they concluded that seeds from wild plants are less abundant in β-elemene compared to commercial seeds (38% vs. 49%, respectively) [22]. This finding is in agreement with higher content of compound of interest found in seeds harvested from plants grown from commercial seeds [20]; hence, not only the plant origin, but what is more more important, the plant cultivation, influence the content of β-elemene in essential oil. According to Wang et al. [1], *N. damascena* essential oil is the richest alternative to *Rhizoma curcuma* source of β-elemene. This source can be used in elemene processing industry delivering β-elemene for antineoplastic medicine. Therefore, we aimed to elaborate the protocol for β-elemene isolation from mentioned essential oil applying HPCCC technique.

Application of high performance counter-current chromatography enabled us to obtain β-elemene of high purity and recovery in 70 min. The previously reported isolation of β-elemene is mostly based on silica gel column chromatography [2] or silver ion coordination chromatography [3,4,5]; however, the latter one is more effective due to the formation of coordination complex between silver ion and the double bond of β-elemene [3]. The formation of coordination complex is, therefore, selective, and enabled the removal of all compounds without double bonds. Wei et al. [5] obtained β-elemene with 56.1% purity using AgNO_3_ silica gel column chromatography; however, they also show that HP20 macroporous resin chromatography gives higher purity of target compound (82.9%). To obtain β-elemene with purity similar to obtained in our study (96%), the additional step of preparative HPLC was required after HP20 column chromatography [5]. There were also a few attempts to isolate β-elemene applying counter-current chromatography. High-performance centrifugal partition chromatography (HPCPC) resulted in elution of β-elemene after 400 min [7], whereas our protocol enabled the elution of target compound in 70 min. Also, silver ion coordination high-speed counter-current chromatography resulted in higher elution time of β-elemene (over 80 min) [8] compared to our method. The differences in the elution time come from different separation conditions between these techniques (HPCCC vs. HPCPC or HSCCC; e.g., rotation speed, column volume, solvent system flow rate); however, they also come from the different essential oils used. In both previously applied counter-current chromatographic techniques, *C. Wenyuj in* essential oil was used for β-elemene isolation. Because *C. wenyujin* essential oil contains a lot of constituents, Lu et al. [8] introduced a silica gel column chromatography step before HSCCC separation. This enabled the removal of impurities and increased β-elemene content in the purified fraction [8]. The two steps separation resulted in around 63% recovery [8]. The isolation protocol described in our study is more effective, since no purification step is required to obtain β-elemene with purity up to 96% and 80% yield. Although *C. wenyujin* essential oil served as material for separation of curdione, curcumol, germacrone, curzerene, 1,8-cineole, and β-elemene [7,25], it is not the most abundant source of β-elemene. In our study, we used *N. damascena* essential oil as more cost-effective source of target compound. The chosen essential oil contains less constituents (29) than *C. wenyujin* essential oil (72) [26], and the content of β-elemene in the first essential oil is at least three times higher, which makes the isolation more effective. The source of β-elemene for industry processing is an extract of rhizome curcuma obtained by means of supercritical CO_2_ extraction [1]; however, it contains smaller amount of compound of interest (up to 27.83%) than source used in our study, and supercritical CO_2_ extraction technique itself is much more expensive and far less effective in β-elemene isolation than simple hydrodistillation, which was proved by Sajfrtova et al. [27]. What is more, the β-elemene injection applied clinically as a national second-class anticancer drug in China contains β-elemene accompanied by its structural isomers, γ- and δ-elemene, and small amount of other terpenoids, among them β-caryophyllene as a main impurity. β-, γ-, and δ-Elemene account for 85% in elemene injection [28]. In our study, we obtained β-elemene of better purity, up to 96%, depending on the amount of sample loaded into column, and we proved the usefulness of *N. damascena* essential oil as a source for β-elemene isolation. To our knowledge, this is the first application of HPCCC technique for β-elemene isolation from the plant material and the first one from *N. damascena* essential oil.

### 3.2. β-Elemene and N. damascena Essential Oil Exert Action against M. tuberculosis H37Ra

The literature data show the activity of many essential oils containing β-elemene (including EO from *C. wenyujin*) against different pathogenic bacteria [29,30,31]; however, the activity of β-elemene itself was studied only by Zhu et al. [31] and our team. In the previous report, β-elemene was active against Gram-positive bacteria *Propionibacterium acnes*, and *Staphylococcus aureus* (strain different than in our study), as well as against fungus *Malassezia furfur* [31]. The results of this study show no activity of β-elemene against tested bacteria with exception of *Mycobacterium tuberculosis* H37Ra. What is more, only our previous work [32], together with this study, describe the influence of β-elemene on *Mycobacterium tuberculosis* and *Mycobacterium smegmatis*. The investigated earlier, isolated strain of *M. tuberculosis* was more sensitive to β-elemene action than that used in the current study H37Ra reference strain; however, the activity of this compound against *M. tuberculosis* is still noticeable, making β-elemene worthy f further testing. Investigated in this study, *N. damascena* EO showed similar activity to β-elemene activity against *M. tuberculosis* and no activity against other microorganisms. The other studies investigating extracts from *N. damascena* seeds and from callus cultures also showed no activity against Gram-positive and Gram-negative bacterial strains and yeast [33,34]. However, Fico et al. [35] described that EO of *N. damascena* inhibited the growth of several bacteria strains in an agar disc diffusion assay. The differences in the observed activity may be explained by different methodology used to evaluate examined samples.

The performed study showed that isolated β-elemene is twofold more potent against *M. tuberculosis* than whole *N. damascena* essential oil, in which β-elemene constitutes almost 50%. It may suggest that the main ingredient is responsible for the activity of EO. Our findings are in agreement with those published previously by Zhu et al., 2013, who also observed that β-elemene is twofold more active against *Propionibacterium acnes* and *Staphylococcus aureus* than *Curcuma wenyujin* EO [31]. This confirms that studied compound is the main one responsible for EO activity. However, the literature data also presents opposite results, suggesting the synergistic action of essential oils constituents, which is expressed in lower MIC values obtained for whole essential oil compared to its main ingredients [36,37].

## 4. Materials and Methods

### 4.1. Plant Material

Commercially available seeds of *Nigella damascena* L. (100 g) obtained from Vilmorin Garden (Komorniki, Poland) were subjected to 3 h hydrodistillation in Deryng apparatus to obtain essential oil [38].

### 4.2. GC-MS Analysis

GC–MS was performed with a Shimadzu GC-2010 Plus coupled to a Shimadzu QP2010 Ultra mass spectrometer (Japan). A fused-silica capillary column ZB-5 MS (30 m, 0.25 mm i.d.) with a film thickness of 0.25 mm (Phenomenex, Torrance, CA, USA) was used. GC-MS conditions applied for analysis of the essential oil and obtained fractions followed Sieniawska et al. [12]. The retention indices were determined in relation to a homologous series of *n*-alkanes (C_8_–C_24_) under the same operating conditions. Constituents of studied essential oil were identified by comparison of their mass spectra and retention indices with computer-supported spectral libraries (Mass Finder 2.1 and NIST database) and with authentic standard of β-elemene.

### 4.3. Isolation and Identification of β-Elemene

Isolation was carried out with Spectrum High-performance counter-current chromatographic (HPCCC) equipment purchased from Dynamic Extraction Co., Ltd. (Slough, Berkshire, UK). The two-phase solvent system suitable for further separation was selected according to the partition coefficient (K) of the target compound (Table 1). Procedure of two-phase solvent system and sample solution preparation was described in detail previously [12]. The separation was performed in the solvent system composed of petroleum ether, acetonitrile, and acetone in a ratio of 2:1.5:0.5 (*v/v*), in the reversed phase mode at 26 °C. The rotation of bobbins applied was 1600 rpm, while the flow rate was 1 and 6 mL/min in the analytical and preparative conditions, respectively. The optimized analytical conditions were scaled up for preparative separation (from 22 mL coil to 137 mL, respectively). The fractions monitored at 210 nm were collected per minute and analyzed by GC-MS. The mass of sample (100, 165, and 200 mg of essential oil) was optimized to obtain the best preparative separation.

Chromatographic (GC profiles) and spectroscopic (mass and NMR spectra) data were used to confirm identity and purity of isolated compound. Bruker Ultrashield 300 spectrometer (Bruker Corporation, Billerica, MA, USA) was used to perform NMR analysis.

### 4.4. Antimicrobial Assay

#### 4.4.1. Tested Organism and Storing Conditions

The following reference bacterial and fungal strains were used in the studies: *Staphylococcus aureus* ATCC 25923, *Staphylococcus epidermidis* ATCC 12228, *Escherichia coli* ATCC 25922, *Pseudomonas aeruginosa* ATCC 27853, *Mycobacterium smegmatis* ATCC 607, *Mycobacterium smegmatis* ATCC 19420, *Mycobacterium tuberculosis* H37Ra ATCC 25177, *Candida albicans* ATCC 10231, and *Candida parapsilosis* ATCC 22019. All microbial strains stocks except mycobacteria were stored in −70 °C in Viabank vials (Medical Wire & Equipment Co. Ltd., Corsham, UK). Mycobacteria were stored in −70 °C in Middlebrook 7H9 broth (Difco Laboratories Inc., Detroit, MI, USA) with 10% albumin dextrose complex (ADC; Becton, Dickinson and Company, Franklin Lakes, NJ, USA) supplemented with 15% glycerol. All microbiological procedures were made according to Clinical and Laboratory Standards Institute [39,40].

#### 4.4.2. Inoculum Preparation

***Mycobacterium tuberculosis***

*M. tuberculosis* H37Ra was grown on Löwenstein-Jensen slopes (BioMaxima, Lublin, Poland) for up to two weeks. Bacterial mass was transferred to 5 mL of the fresh Middlebrook 7H9 broth supplemented with 10% ADC and vortex with 1 mm glass beads for 3 min. After 30 min of incubation (lager clumps sedimentation) in room temperature, upper 2 mL was transferred to a sterile tube and left for next 15 min. One milliliter of supernatant was placed in a sterile tube and was adjusted to 0.5 McFarland standard with ADC-supplemented Middlebrook 7H9 broth. The density of bacterial suspension used for the plate inoculation was 1 × 10^6^ CFU/mL.

***Mycobacterium smegmatis***

The inocula of *M.* *smegmatis* were prepared in the same way as *M.* *tuberculosis*. Instead of the Middlebrook 7H9 with ADS, Mueller-Hinton Broth (MHB) (BioMaxima, Lublin, Poland) was used.

**Other bacterial strains**

The inocula of all other bacterial strains used in this study were prepared with the direct colony suspension method in MHB. Colonies from 24 h Mueller-Hinton Agar (MHA)(BioMaxima, Lublin, Poland) plate were scraped, suspended in MHB, and adjusted to the turbidity of 0.5 McFarland standard. The density of bacterial suspension used for the microtiter plate inoculation was 1 × 10^6^ CFU/mL.

**Yeasts**

Colonies of approx. 1 mm in diameter were picked from 24 h Sabouraud dextrose agar plate (BioMaxima, Lublin, Poland), suspended in 5 mL of sterile 0.85% saline, and vortexed for 15 s. The cell density was adjusted to 0.5 McFarland standard with sufficient volume of sterile saline. Resulted suspension was diluted with RPMI 1640 broth medium to obtain twofold test inoculum 5 × 10^3^ CFU/mL used to inoculate microtiter plate.

#### 4.4.3. Dilutions of Tested Substances

*N. damascena* essential oil and β-elemen were tested in the concentration range 1000–32 µg/mL. Serial twofold dilutions were prepared in dimethyl sulphoxide (DMSO; Sigma-Aldrich, St. Louis, , MO, USA) and diluted with medium suitable for the given organism. The final DMSO concentration in the well did not exceed 1% (*v/v*) and had no influence on growth of the tested strains.

#### 4.4.4. References Antimicrobials

Ciprofloxacin, gentamicin, cefoxitin, amphotericin B, and ethambutol (Sigma-Aldrich, St. Louis, MO, USA) were used as reference standards. Stock solutions were prepared according to the manufacturer’s instructions. Final twofold dilutions from 16 to 0.004 μg/mL were prepared in broth suitable for given microbial.

#### 4.4.5. Plates Preparation

The round bottom microwell plates were prepared as follows: in each well, 50 μL of inoculum and 50 μL of tested substances were combined. The final density of inoculum in each well was approx. 5 × 10^5^ CFU/mL for all bacterial strains and approx. 2.5 × 10^3^ CFU/mL for yeast strains. The sterility, growth, and 1% DMSO controls were carried out for each tested strain. The plates were closed with sealing foil to prevent liquid evaporation and incubated at 35 °C except *M. smegmatis* incubated at 29 °C.

#### 4.4.6. Determination of Minimal Inhibitory Concentration (MIC)

**The resazurin microtiter assay for *Mycobacterium tuberculosis***

After 8 days of incubation, 10 µL of resazurin (alamarBlue; Invitrogen, Carlsbad, CA, USA) solution was added to each well, incubated 48 h at 35 °C, and assessed for color development. A change from blue to pink indicates reduction of resazurin to resorfurin and proofs bacterial viability. The MIC was defined as the lowest drug concentration that prevented this color change [41]. The assay was performed in triplicate.

**The MIC for other microbials**

The MIC was read as follows: *E. coli*, *P. aeruginosa*, *S. aureus*, and *S. epidermidis* after 18 h of incubation; yeasts after 48 h of incubation and *M. smegmatis* after 72 h of incubation. The MIC was defined as the lowest concentration of antimicrobial agent that completely inhibits growth of the organism in the microtitration plate’s wells detected by the unaided eye. The assay was performed in triplicate.

## 5. Conclusions

In this work, *N. damascena* essential oil was shown to be a new, abundant source of β-elemene. The high purity β-elemene was successfully isolated from this EO for the first time. High-performance counter-current chromatography was proved to be an efficient technique for this sesquiterpene separation, while antimicrobial testing revealed moderate activity of β-elemene against *Mycobacterium tuberculosis* H37Ra.

## Figures and Tables

**Table 1:** The chemical composition of *N. damascena* L. essential oil.

No	Compound	tr	RI	Area (%)
**1**	2,4-Dimethyl-heptane	4.057	850	0.16
**2**	Hexanoic acid, izopropyl ester	9.838	1020	0.13
**3**	2-Methoxyl-6-antranilate	13.270	1227	0.10
**4**	Sativen	15.500	1352	0.07
**5**	Longifolene	15.910	1376	0.09
**6**	Iso-β-elemene	16.120	1383	0.13
**7**	β-Bourbonene	16.187	1388	2.38
**8**	β-Elemene	16.270	1398	47.37
**9**	Isocaryophyllene	16.570	1415	0.08
**10**	7-Isoprenyl-1-methyl-4-methylenedecahydroazulene	16.707	1428	0.91
**11**	β-Caryophyllene	16.803	1430	0.06
**12**	Selina 4,11-diene	17.640	1482	2.68
**13**	*γ*-Gurjunene	17.703	1486	0.69
**14**	Valencene	17.787	1488	0.69
**15**	β-Selinene	17.940	1495	10.10
**16**	*α*-Selinene	18.043	1506	13.52
**17**	*α*-Bulnesne	18.143	1514	0.43
**18**	8-Isopropenyl-1,5 dimethyl 1,5-cyclodecadiene	18.223	1519	1.18
**19**	*δ*-Cadinene	18.320	1524	0.16
**20**	7-Epi-*α*-selinene	18.403	1532	3.26
**21**	Metyl 2-amino-3-methoxyl benzoate	18.780	1553	0.06
**22**	Elemol	18.807	1557	0.93
**23**	Damascenine	19.073	1575	11.97
**24**	Elema-1,3-dien-6-α-ol	19.837	1630	0.24
**25**	Selina-6-en-4-ol	20.523	1673	0.67
**26**	Longifolenaldehyde	20.670	1680	0.07
**27**	Oleylalkohol, metyl ether	22.300	1802	0.22
**28**	Unknown	22.353	1807	1.07
**29**	1-Octadecanol, metyl ether	22.623	1828	0.32
	Total			99.2

tr—retention time, RI—measured retention indices.

**Table 2:** The partition coefficients values for β-elemene in different solvent systems.

No	Solvent Systems (*v/v*)	K
**1**	Heptane/Acetonitrile/Ethyl acetate (2:1:1) *	1.54
**2**	Petroleum ether/Acetonitrile/Acetone (7:6:1)	4.46
**3**	Petroleum ether/Acetonitrile/Acetone (2:1:1) *	1.74
**4**	Petroleum ether/Acetonitrile/Acetone (2:1.5:0.5)	2.58

K—partition coefficient; * the solvent system becomes a single phase above 23°C.

**Table 3:** The influence of sample mass on the purity and yield of β-elemene.

Mass of Sample	Fraction (min)	Purity (%)	Yield (mg)
200 mg	49–56	87	90
	57–61	92	22
165 mg	50–58	86	78
	59–64	92	25
100 mg	55–62	89	47
	63–68	96	22
	69–70	92	11

**Table 4:** The antimicrobial activity of tested samples. The activity was expressed as minimal inhibitory concentration (μg/mL).

	CIP	GEN	FOX	ETB	AMP	ND	E
*Staphylococcus aureus* ATCC 25923	0.25	0.25	-	-	-	>1000	>1000
*Staphylococcus epidermidis* ATCC 12228	0.06	0.125	-	-	-	>1000	>1000
*Escherichia coli* ATCC 25922	0.008	0.5	-	-	-	>1000	>1000
*Pseudomonas aeruginosa* ATCC 27853	0.25	2	-	-	-	>1000	>1000
*Candida albicans* ATCC 10231	-	-	-	-	0.03	>1000	-
*Candida parapsilosis* ATCC 22019	-	-	-	-	0.25	>1000	-
*Mycobacterium smegmatis* ATCC 607	0.25	-	16	-	-	>1000	>1000
*Mycobacterium smegmatis* ATCC 19420	0.25	-	16	-	-	>1000	>1000
*Mycobacterium tuberculosis* H37Ra ATCC 25177	0.125	-	-	2	-	256	128

CIP—Ciprofloxacin; GEN—Gentamicin; FOX—Cefoxitin; AMP—AmphotericinB; ETB—Ethambutol; ND—*Nigella damascena* EO; E—β-elemene. Drugs were used as positive controls; 1% DMSO was used as a negative control.

## Supplementary Materials

Supplementary materials are available online. Figure S1: HPCCC chromatogram of N. damascena essential oil, Figure S2 A: GC chromatogram of N. damascena essential oil, B: GC chromatogram of β-elemene standard, C: GC chromatogram of isolated β-elemene, D: MS spectrum of β-elemene; Figure S3: ^1^H NMR spectrum of β-elemene; Figure S4: ^13^C NMR spectrum of β-elemene.
