# Supplementary material for: Nigella damascena L. Essential Oil—A Valuable Source of β-Elemene for Antimicrobial Testing
Source: Molecules. 2018 Jan 28;23(2):256. doi: 10.3390/molecules23020256 (PMC6017462; doi:10.3390/molecules23020256)
Supplement: Supplementary file 1 [file molecules-23-00256-s001.pdf]

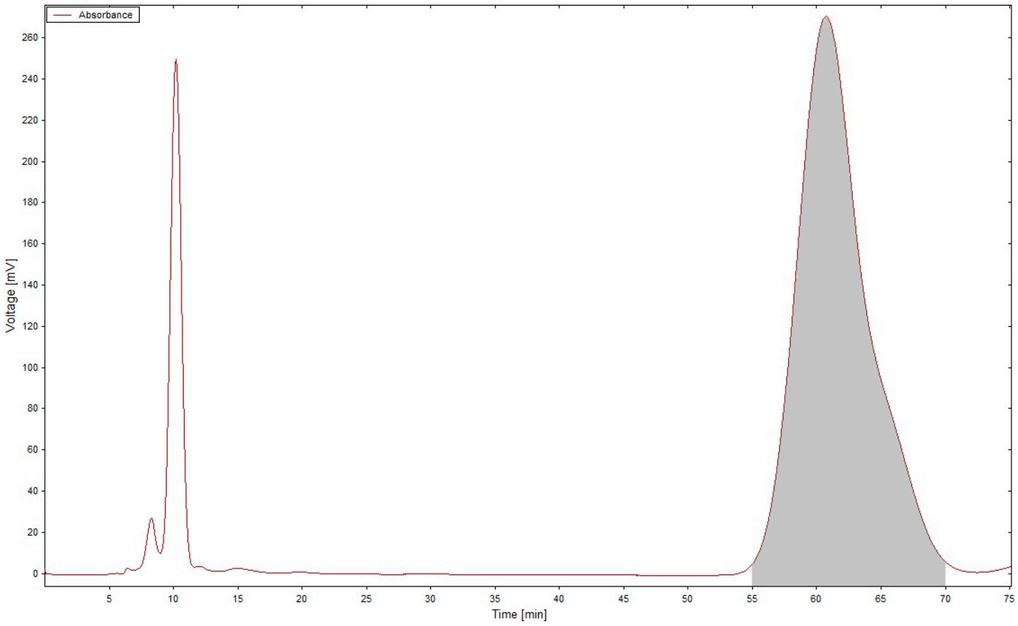

Figure S1. HPCCC chromatogram of *N. damascena* essential oil.

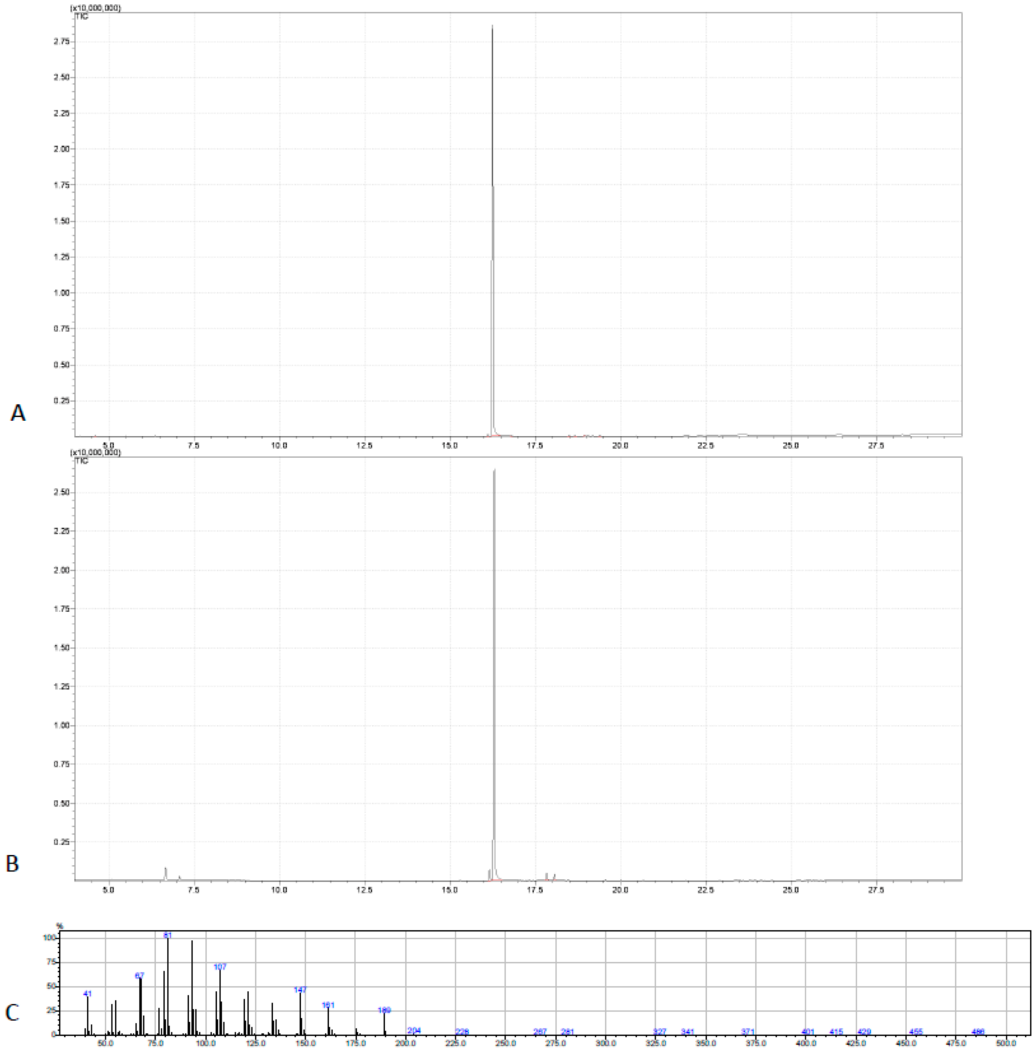

Figure S2 A: GC chromatogram of *N. damascena* essential oil, B: GC chromatogram of β-elemene standard, C: GC chromatogram of isolated β-elemene, D: MS spectrum of β-elemene;

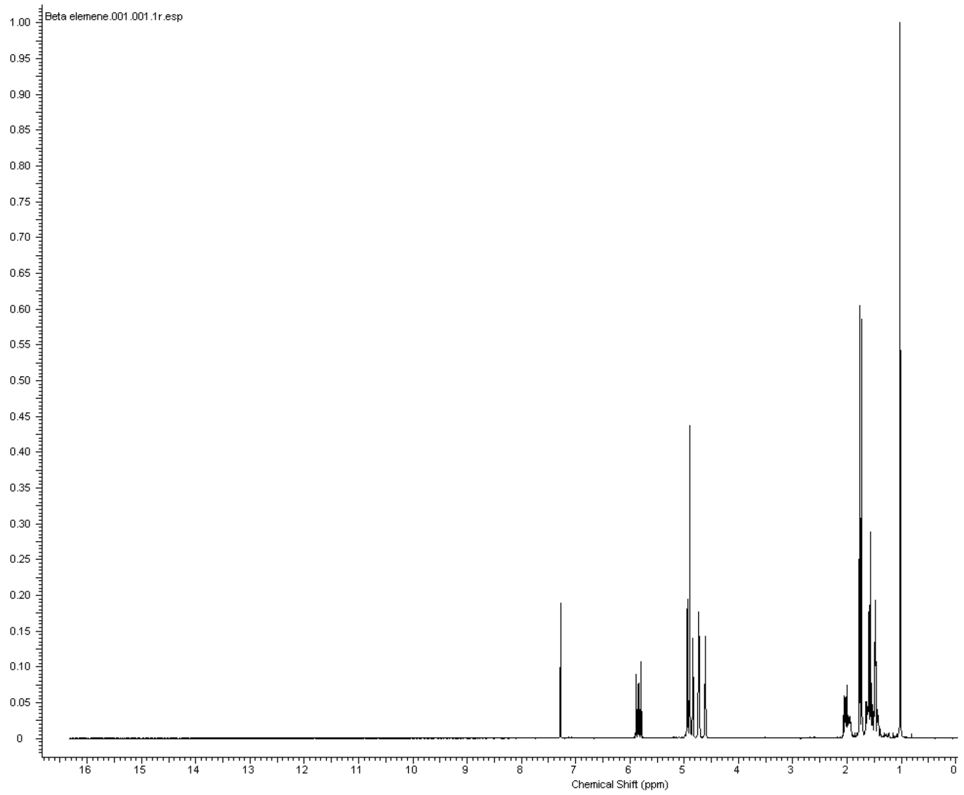

Figure S3: ^{1}H NMR spectrum of β-elemene.

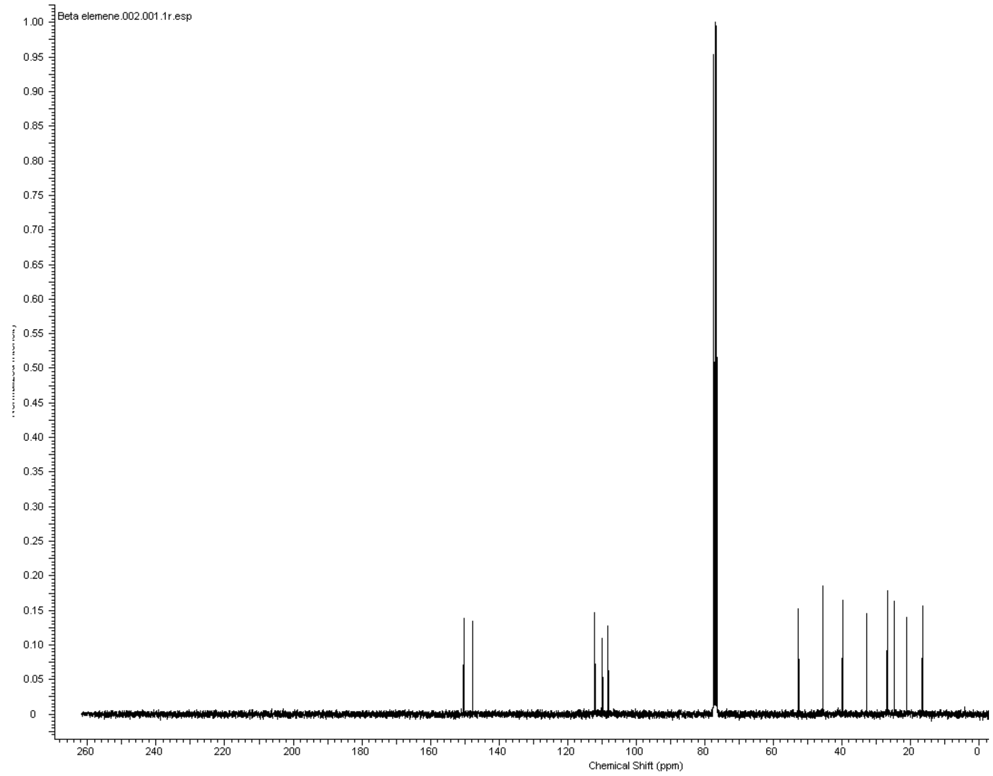

Figure S4: ^{13}C NMR spectrum of β-elemene.
